# A CNN based m5c RNA methylation predictor

**DOI:** 10.1038/s41598-023-48751-9

**Published:** 2023-12-11

**Authors:** Irum Aslam, Sajid Shah, Saima Jabeen, Mohammed ELAffendi, Asmaa A. Abdel Latif, Nuhman Ul Haq, Gauhar Ali

**Affiliations:** 1 Department of Computer Science, COMSATS University Islamabad, Abbottabad Campus, Abbottabad, 22060 KPK Pakistan; 2 EIAS Data Science Lab, College of Computer and Information Sciences, Prince Sultan University, Rafha, Riyadh, 12435 Saudi Arabia; 3 College of Engineering, AI Research Center, Alfaisal University, Riyadh, 50927 Saudi Arabia; 4 Public Health and Community Medicine Department (Industrial medicine and occupational health specialty, Faculty of Medicine, Menoufia University, Shibîn el Kôm, Egypt

**Keywords:** Computational biology and bioinformatics, Epigenetics, Epigenomics, Computer science

## Abstract

Post-transcriptional modifications of RNA play a key role in performing a variety of biological processes, such as stability and immune tolerance, RNA splicing, protein translation and RNA degradation. One of these RNA modifications is m5c which participates in various cellular functions like RNA structural stability and translation efficiency, got popularity among biologists. By applying biological experiments to detect RNA m5c methylation sites would require much more efforts, time and money. Most of the researchers are using pre-processed RNA sequences of 41 nucleotides where the methylated cytosine is in the center. Therefore, it is possible that some of the information around these motif may have lost. The conventional methods are unable to process the RNA sequence directly due to high dimensionality and thus need optimized techniques for better features extraction. To handle the above challenges the goal of this study is to employ an end-to-end, 1D CNN based model to classify and interpret m5c methylated data sites. Moreover, our aim is to analyze the sequence in its full length where the methylated cytosine may not be in the center. The evaluation of the proposed architecture showed a promising results by outperforming state-of-the-art techniques in terms of sensitivity and accuracy. Our model achieve 96.70% sensitivity and 96.21% accuracy for 41 nucleotides sequences while 96.10% accuracy for full length sequences.

## Introduction

In the current era we are swimming in an extending sea of information. Data with big volume, high velocity, and variety is obtained from various fields of sciences and engineering^1–3^. Life researchers are also going to grapple with massive data because of high-throughput genomics. They are facing vast range of problems related to handling, processing, storing and interpreting biological data. The techniques used to generate biological data, spit out various types of information, such as interactions of proteins, genomic sequences or findings in medical records etc. Since, the biological data come from wide range of methods, that is why when compared to other domains of science it is highly heterogeneous in nature.

Learning from big sets of data (massive data) is highly challenging but undoubtedly it is the essential part of numerous fields in the current time. It needs new ways of thinking to acknowledge the challenges of learning with massive data and the related convenient solutions. The novel techniques of learning were required which possess the ability to fully making sense of big data. In other words we need the algorithms which are inherently efficient and powerful to tackle the data which poses the challenges due to its high dimensions, imbalance, heterogeneous and uncertain nature^4,5^.

The amount of biological sequential data has also increased in the last decade with the advent of high-throughput sequencing projects. A biological sequence data is a continuous and single string or molecule of protein or nucleic acid which are made from amino acids or nucleotides respectively. The amino acids, nucleotides and ribonucleotides are the basic structural and functional blocks or units of the three fundamental and informative life’s polymers that is proteins, DNA and RNA respectively.

The genetic information present in DNA molecule is interpreted and copied by different types of RNA polymerases. A specific well defined sites of DNA is decoded and transcribed into a variety of single-stranded transcripts (RNAs). Furthermore, there are four standard ribonucleotides i.e., U, A, G and C involved in the production of RNA molecules. Ribonucleic acid (RNA) is the main polymeric molecule that transfer instructions from genes to ribosomes in order to synthesize specific proteins. Each triplet codon within transcript e.g mRNA has been translated into an appropriate and relevant amino acid of protein chains. There are 21 standard amino acids which are categorized as essential and non-essential entailed in the synthesis of protein molecules.

However, in every biopolymer type, the limited amount of basic building blocks seems to be necessary for the natural flow of biological information from DNA to RNA to protein. Although it appears that they are not sufficient to attain all expected and anticipated functions of these polymers in organisms. DNA, RNA and proteins are composed only from few basic units, so in order to perform such a variety of functions some targeted enzymes have altered the units at specific locations to produce new characteristics of molecules. The modifications in polymer molecules are termed as pre or post replicational , transcriptional or translational changes. Due to these modifications, the biopolymer got new functional or structural features thus allow them to perform more complicated functions in a well-organized manner^6–10^.

**Figure 1:**
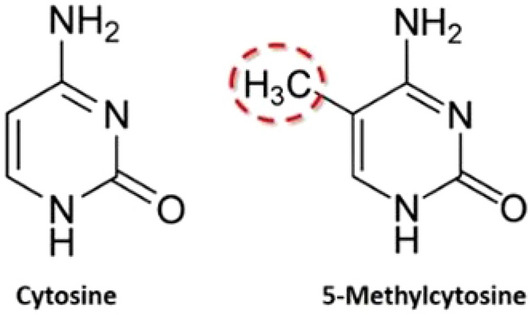
Cytosine with and without methylation.

Post-transcriptional modifications of RNA play a key role in performing a variety of biological processes, such as stability and immune tolerance, RNA splicing, protein translation and RNA degradation. One of these RNA modifications is m5c which participates in various cellular functions like RNA structural stability and translation efficiency, got popularity among biologists. More specifically, m5C modification also known as methylation occurs at $5\textrm{th}$ position of cytosine when methyl group ($CH_3$) is added to it as shown in Fig. 1. A comprehensive study is presented in^11^ about the implication of RNA m5c modification in cancer.

By applying biological experiments to detect RNA m5c methylation sites would require much more efforts, time and money^12^. To know the logic of life it is essential to interpret the full spectrum of m5c methylation and its position in the RNA molecule. The m5c modified RNA molecules can be enunciated or presented as genetic or physical map, an actual sequence of amino acids or nucleic acids, or some more complicated data representation. To get insight into methylated molecule’s function, there is a need to analyze hidden features with in these modified molecules.

In order to explore the hidden features in data, feature extraction techniques are widely used in data analysis field. Feature extraction refers to identifying an interpretable and discriminating representation of data for machine learning models that can enhance the prediction power of classifier and its performances. The performances of underlying classifier depends on the quality of extracted features.

Feature extraction can either be handcrafted or automated, depending on the nature of the problem, the amount of data and available resources. The manual feature extraction known as hand crafted features is not only difficult and time consuming process but also some time these features may not effectively represent the underlying objects/entities (sequences in our case). Apart from the above challenges, fully domain expertise is also required to carry out this task. Now a days large amount of data is available due to which both biologists and computer scientists are confronted with many difficulties to speedily perform data analysis tasks in life sciences. In addition to scalable and efficient methods, high performance computing (HPC) platforms and automatic feature extraction techniques are entailed to gain a keen insight into the biological functions from big data. The key feature of deep learning techniques is representation learning which extracts a diverse range of meaningful descriptors/ features that enhance the prediction capabilities of the underlying model. By using this approach, feature extraction and classification can be done in an end-to-end manner, enabling us to obtain the significant high level features automatically, resulting in improved performances^13–17^.

Since further improvements have been enabled by the use of greater computational resources, especially graphics processing units (GPU), allowing training of deep networks containing various parameters in an appropriate time. It allows us to efficiently train specialized deep networks such as convolutional neural networks (CNN) and recurrent neural networks (RNN) with long short-term memory cells (LSTM). These networks have been successfully applied to many problems including image recognition and natural language processing tasks like language translation and speech recognition^18–20^.

Prediction of m5c poses some of the challenges. For example, the nucleotide preference around m5c cite is not known. So lack of clear sequence context information of m5c cite would led to intricacy in the prediction method. It may possible that the motif of m5c is obscured so it is difficult to find local sequence context of m5c cites. Most of the researchers are using pre-processed RNA sequences of 41 nucleotides, therefore, it is possible that some of the information around these motif may have lost. The conventional methods are unable to process the RNA sequence directly and thus need optimized techniques for better features extraction. Being high dimensional, the methylated datasets usually posed a challenge to conventional analysis techniques. It is also termed as curse of dimensionality.

To handle the above challenges the goal of this study is to employ an end-to-end deep learning model as a powerful toolbox to classify and interpret m5c methylated data sites. The power of deep learning models for high dimensional data is proven in literature. Moreover, our aim is to analyze the sequence in its full length where the methylated cytosine may not be in the center. Thus, the contribution of this work to use analyze the sequences in a more natural way (closed to reality) using an end-to-end deep learning model to automatically extract the features.

We have obtained state-of-the-art results for both 41 nucleotides and full length datasets (see Table 3. This paper is organized as follows: Related work is discussed in section “Related work”. Our proposed model is presented in section “Materials and methods”. The obtained results are discussed in section “Results and discussion” and finally the paper is concluded in in section “Conclusions”.

## Related work

Methyl group attachment to RNA is a type of post-transcriptional modification controlling the mechanism of RNA interaction with other components of the cell. Recent research have been linked the RNA modifications to different processes ranges from alternative splicing to various diseases, including cancer. Understanding RNA modifications will let a new level of fine tuning of gene expression. It will have a significant impact on various field like fundamental biology, biotechnology, medicine and crop production etc.

In this section we mainly discussed only experimental or machine learning techniques which have been done previously on RNA methylation detection and classification.

The selection of experimental methods have shown in Table 1, depends on the type of modification, its abundance and pre-existing knowledge of context in the modified sequence^21,22^. Furthermore, these techniques are expensive in terms of time and money.

**Table 1:** Strategies for the detection of RNA methylation.

Sr.	Experimental methods
1	Radioisotope incorporation^21^
2	Thin-layer chromatography^23^
3	Mass spectrometry^21^
4	Differential enzyme or Chemical-RNA interactions^21^
5	Bisulphite RNA sequencing^21^
6	Antibody-based sequencing^21^

Many computational tools have been built due to the rapid developments of bioinformatics and machine learning techniques^24,25^. Considering the importance of RNA methylation specifically the m5c, there have been many computational tools designed till date that are used to detect or identify m5c RNA methylation. The developed tools or proposed approaches mainly worked on the primary sequence of RNA.

**Figure 2:**
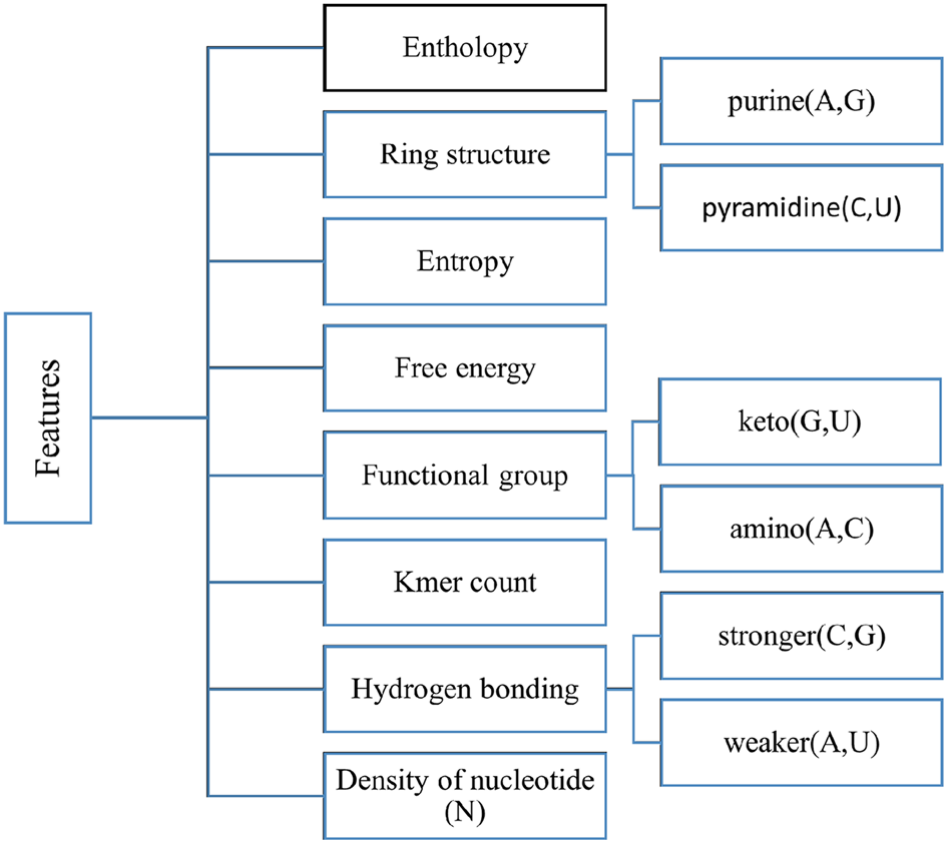
Taxonomy of features.

Three vital steps are used in the development of m5c methylation predictor: (1) data collection, (2) feature extraction, and (3) classification or prediction. The taxonomy of features which are used as input to machine learning models are given in Fig. 2.

A number of computational methods as shown in Table 3 had been developed to predict RNA m5c methylation. The description of these methods is discussed in this section.

Pengmian Feng et al.^26^, used a support vector machine based-method to predict m5c sites in homo sapiens transcriptome. In the proposed method, RNA sequences were encoded applying the pseudo dinucleotide composition in which three RNA physiochemical features were incorporated. It was observed that the overall success rate that is gained by the developed model is 90.42%.

Pengmian Feng et al.^27^ proposed a classification method that was applied for the classification of three kinds of RNA modification m1a, m6a and m5c. Local features (ring structure, functional group and hydrogen bonding) and density information of nucleotides have been employed to encode a RNA sequence. The encoded sequence is converted into general pseudo K-tuple nucleotide composition (PseKNC) vector which is used to train SVM based predictor called iRNA-PseColl. The classifier achieved 77.50% classification accuracy on a human transcriptome m5c methylated dataset.

The authors in^28^ proposed a new predictor called iRNAm5C-PseDNC, which has been developed by embodying ten different types of physical-chemical features into pseudo dinucleotide composition through the auto/cross covariance technique. Rigorous jackknife tests has been used which shown that the anticipated accuracy of predictor is 92.37%.

In^29^ the researchers built a model for the prediction of m5c cites. The proposed model based on composite features in which three features extraction techniques were combined. After feature extraction, MRMR (Minimum Redundancy Maximum Relevance) was applied as a feature selection method and SVM was used as classifier. The dataset used in this study acquired from RM-base data base in which each sequence is 41 nucleotide long and methylated cytosine is positioned in the center. The predictor have an accuracy up to 93.33%.

In^30^ a new m5c site predictor called M5C-HPCR is proposed in which multiple base classifiers are properly integrated in order to improve the accuracy of classification tasks. This combination of multiple classifiers in this manner is called ensemble classification. In order to get discriminative features and encoding method has been done by applying a heuristic nucleotide physicochemical property reduction algorithm(HPCR). The predefined algorithm extracts multiple redacts of physical-chemical properties which were used as input for ensemble classifier. They have demonstrated results for two benchmark dataset using jackknife test and got MCC = 0.850 and AUC = 96.2%.

In^31^ RNAm5Cfinder, a web-server developed based on random forest algorithm. It is an efficient tool uses RNA sequence features to identify RNA m5c sites in eight different cell types from mouse and human. The results show that the cell-specific predictors could perform better. For the tissue-specific m5c sites prediction in human the obtained, area under curve (AUC) is 77%.

In^12^ a transfer learning based deep model DeepMRMP was built to predict different types of RNA modification sites. They have predicted multiple RNA site modifications for three species i.e., H. sapiens, M. musculus and S. cerevisiae. It is one of the reliable tool for predicting N1-methyladenosine (m1A), pseudouridine ($\Psi$) and 5-methylcytosine (m5c) modification sites. The designed predictor had achieved an accuracy up to 66% for m5c data set which is very less.

Dou et al.^32^ have worked on multiple sequences using SVM and other machine learning techniques for arabidopsis thaliana. Chai et al.^33^ have proposed a computational method called staem5 for m5c prediction of mus musculus and arabidopsis thaliana. A deep learning based ensembler classifier is used to predict N5-methylation in^34^. A CNN (Convolutional Neural Network) based model is proposed for prediting different kinds of RNA modifications in^35^.

## Materials and methods

### Dataset

We have used the dataset of Squires et al.^36^ for training and evaluation of proposed model. The used data denoted the widespread occurrence of modified cytosine throughout Human transcriptome^36^. The ensemble transcript IDs were available for each methylated and nonmethylated transcript’s sequences.

The sequences for these IDs were obtained by applying the biomart tool. The tool is supported by ensemble genome browser. The tool provided many functions for obtaining sequence related information. The cDNA (Complementary DNA) for all transcripts have been downloaded from ensemble genome browser^37–39^. cDNA is similar to RNA, the presence of thymine (T) in place of Uracil (U). By simple T$\rightarrow$U transition, the cDNA sequence is transformed into corresponding RNA sequence^40^.

Apart from the whole transcript length we also trained the model on the dataset of corresponding data acquired from RM-base data base in which each RNA sequence is 41 nucleotide long and methylated cytosine is positioned in the center. This dataset is used by almost all research works mentioned in section “Related work”. Redundant sequences were removed using CH-HIT^41^.

### Encoding data

All the sequences were first converted into k-mers by using overlapping sliding window. The selection of value for k in kmer is a strenuous process. Its value varies according to research domain. Basically, the sequence length, equals to L yields (L−k+1) total k-mers, and generate total $n^k$ possible unique k-mers. Here, “n” is number of monomers which is four “U,A,C and G” in case of RNA^42,43^. On the basis of preceded work the proposed research has been selected the value of 3 for k. All the sequences were converted into 3-mers, so according to the formula we have got total 64 unique 3-mers. After obtaining k-mers the next step is to transform the k-mers into a digital vector. It is one of the fundamental phases in the process of feature learning and data representation, because machine learning models require numeric data.

In sequence analysis one-hot encoding is one of the common and effective encoding method, which map each sequence to a digital vector. One-hot digital vector designated every word as a |*V*| dimensional vector with single “1” and the remaining “0s”. Here, |*V*| indicates the size of predefined vocabulary. For example each and every mono nucleotide in RNA can be encoded into a four-dimensional matrix or vector such as A = [1,0,0,0], C = [0,1,0,0], G = [0,0,1,0], U = [0,0,0,1]. So, in our proposed method each 3-mer was converted into a 64 dimentional one-hot digital vector as done by^44–47^. One-hot encoding is the simplest of all encoding techniques. There are advanced encoding techniques like whistle used in^48^, Gene2vec^49^, Geo2vec^50^, Genomics features^48^ etc, but we have selected one-hot encoding because our main focus is to propose a powerfull deep learning classifier which rely less on the underlying encoding technique.

### Data preparation for variable length input sequences

The input sequences are of variable length, so zeros are added (Zero padding) to each sequence up to a required longest common length. In this case the model will automatically learn that zeros carried no information, and they are added to generate same length vector^51^.

### Imbalance data

**Figure 3:**
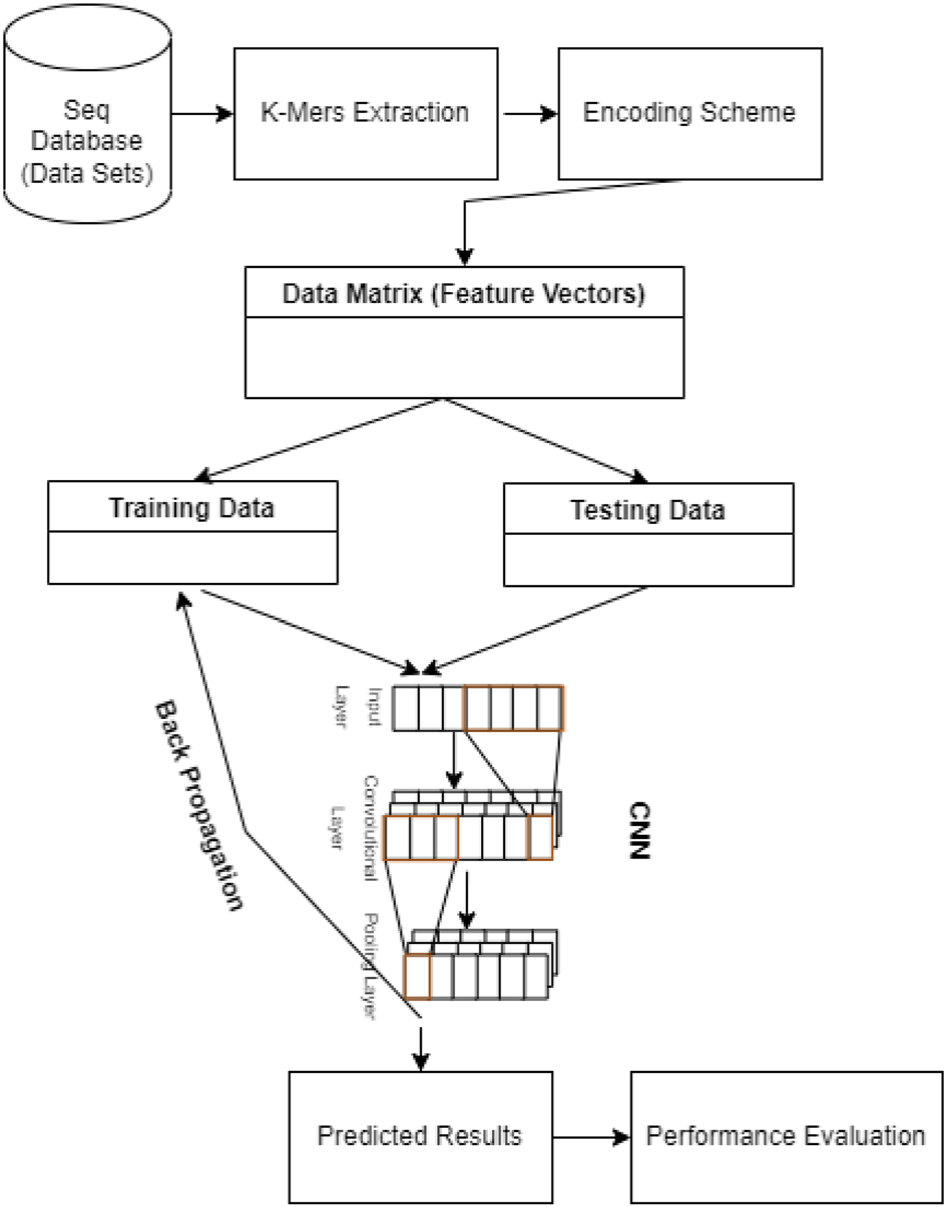
The over all methodology.

The biological data are usually imbalanced in which the negative class out numbered the positive one. It might yield awful results to train a model with such an imbalanced data. In order to overcome this issue, the proposed work relied on weighted cross entropy instead of simple loss function. The weighted loss penalizes the classifier if its performance is not well on the minor class. There are a lot of methods that can be applied to alleviate the issue of imbalance^52–58^. To handle the issue of imbalance data, Yang et al.^52^ has devised a technique called sample subset optimization. The authors in^53^ have proposed a level wise strategy to handle this issue. In^54^ the researchers have used a technique called maximum-AUC to handle imbalanced data. A detailed survey is presented about techniques handling imbalance data in^55^. The authors in^56^ have used NCR (neighborhood cleaning rule) to tackle the issue of imbalance data. The work in^57^ presents a python package while^58^ discusses a hybrid data level sampling technique to handle imbalance datasets. The data is splited into training, validation and testing sets in order to validate the generalization of our model(s). The over all methodology is given in Fig. 3.

### Performance metrics or evaluation indicators and hyper-parameters settings

**Figure 4:**
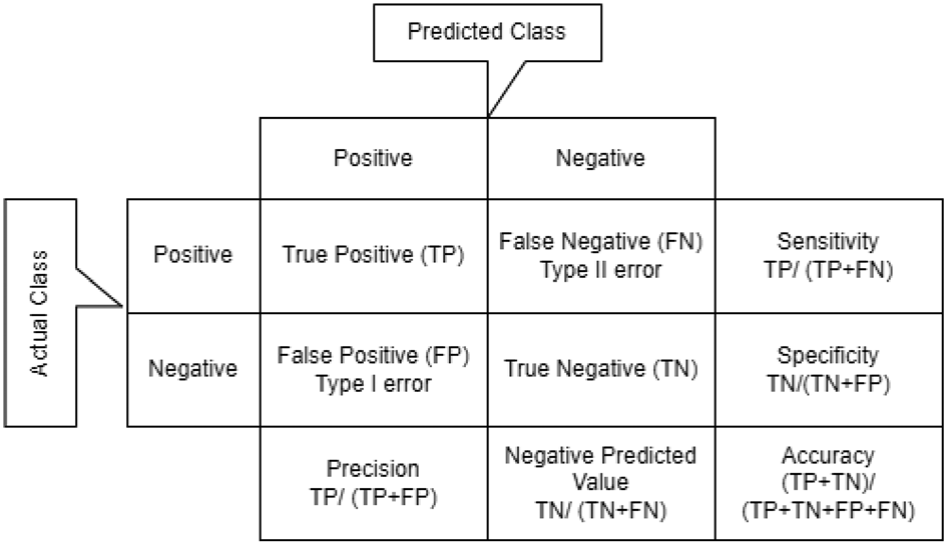
Evaluation metrics.

To evaluate the efficiency of classifier, many metrics were used including specificity ($S_p$), sensitivity ($S_n$), overall accuracy (ACC) and Area Under the Receiver Operating Characteristic Curve (AUC)^59^. The sequences containing methylated sites have been considered as the positive samples and the non methylated ones are negative samples, and all the metrics have computed according to the Formulas shown in Fig. 4. Different hyper-parameters for example learning rate, batch size, activation function, and dropout rate have been encountered. The proposed method has been maintained the default settings of almost all hyper-parameters.The value for the batch size has been selected up to 100. In order to overcome overfitting early stopping was adopted^60,61^.

### Our proposed models

Kunihiko Fukushima first proposed CNN in 1988^62^. There are three variants of CNN i.e., 1D, 2D and 3D CNN. In our work we have used 1D convolutional neural network, because it is best suited (among the three variants) for sequential data analysis. A detailed and comprehensive review article about the applications of 1D CNN is presented in^63^. Kiranyaz et al.^64^ used 1D CNN for the first time in 2015, on patient-specific ECG signals.

The basic idea to design classifier or network is to used variable size multichannel convolution layers^65,66^. Different sizes of convolution kernels are used to convolve the input data simultaneously at different resolutions or different n-grams (groups of words). Different sizes of convolution kernels increase diversity in the extracted features. The outputs of these layers are concatenated. Global max-pooling^67^, have been applied after convolution. The purpose of max-pooling layer is to focus on the most active or important features in each feature map.

**Figure 5:**
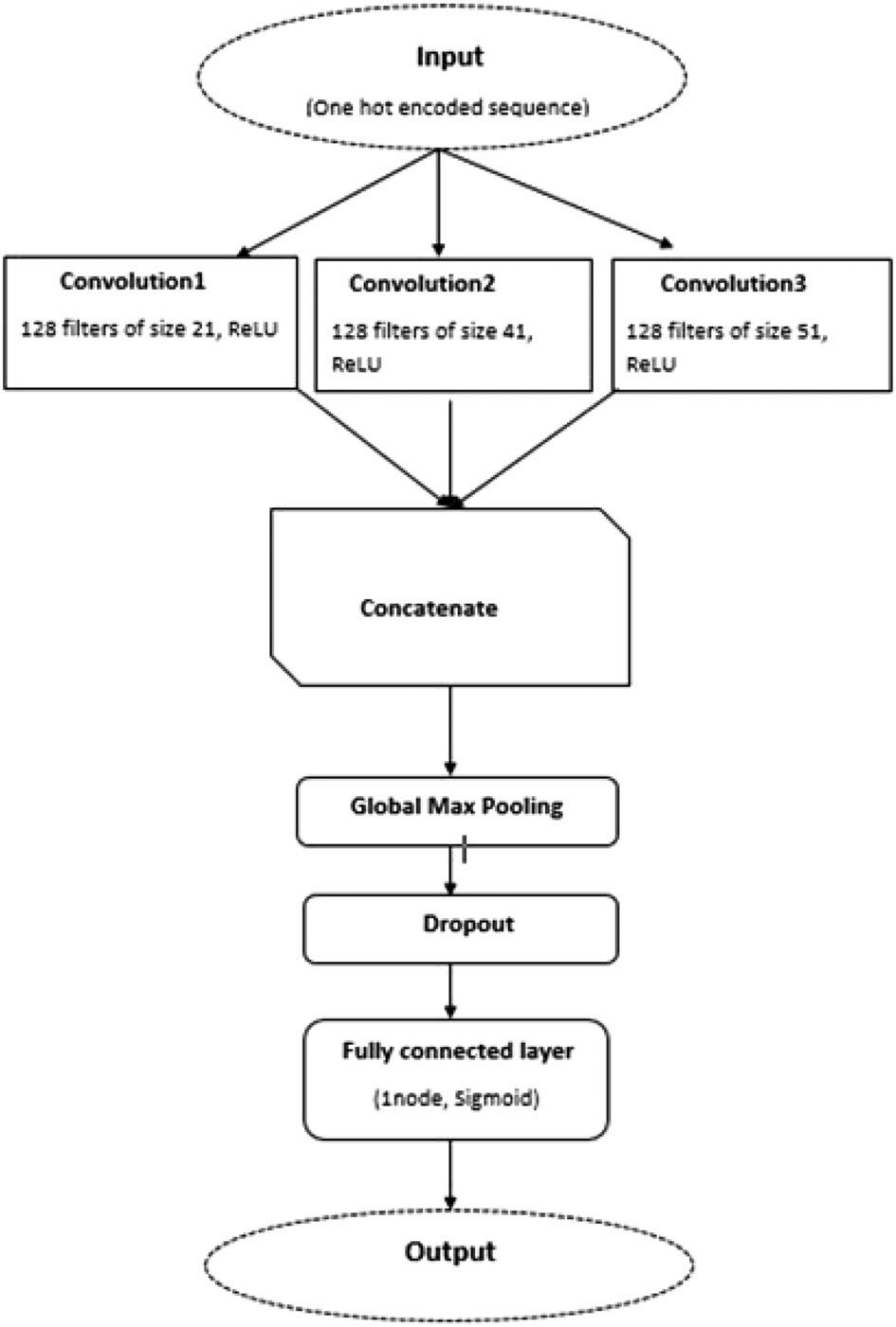
Model Architecture.

In the proposed model three convolutional layers followed by a global max-pooling, followed by a dropout layer were added. At the end a dense layer with one unit as output layer was used. The purpose of dropout layer is to overcome the issue of overfitting. All the layers parameters are shown in Table 2.The proposed model architecture is shown in Fig. 5.

**Table 2:** Classifier parameters.

Layers	Parameters
Input	Sequence length = 5000 and 41, dimension = 64;
Convolution layer1	Filters = 128; Filter-length = 21; Activation = relu
Convolution layer2	Filters = 128; Filter-length = 41; Activation = relu
Convolution layer3	Filters = 128; Filter-length = 51; Activation = relu
Concatenate	[Convolution layer1, Convolution layer2, Convolution layer3]
Global max pooling	–
Dropout	0.5
Output	Activation = sigmoid

## Results and discussion

Using a CNN architecture as the one described earlier we analyzed methylated and non methylated sequences for human. We run the model both on the whole transcript as well as on 41 nucleotide lengthy sequences. The ratio of training and testing dataset is set to 90:10.

**Figure 6:**
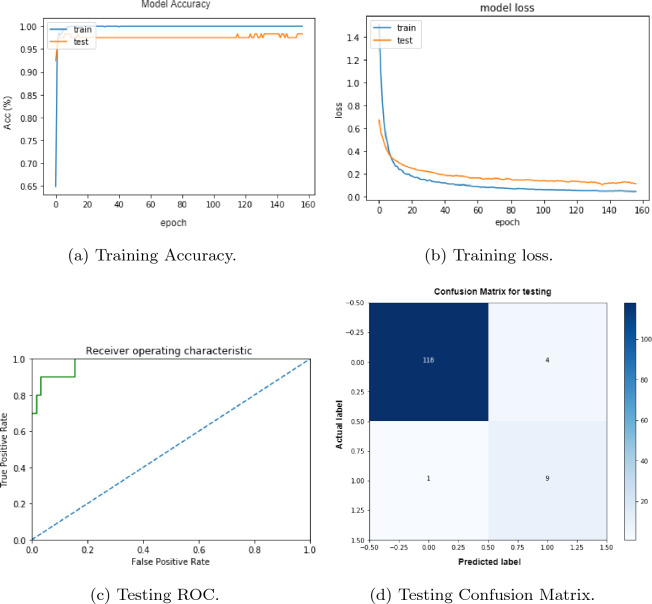
Performance of our model for training/testing data for 41 nucleotide sequences.

The accuracy, loss function’s graph, for training while ROC and confusion matrix for testing are show in Fig. 6 respectively. These experiments were performed on 41 nucleotides lengthy sequences. We compare our model’s results to the state-of-the-art classifiers in Table 3. These works also have used the same dataset of Human. The comparison is based on four different measures accuracy, sensitivity, specificity, and AUROC (see Table 3). For the human dataset taking 41 nucleotide our model shows prediction accuracy up to 96% for testing and 98% for training, outperforming all the methods.

These results demonstrate the ability of deep learning to extract the most significant patterns that characterize the different sequences. Although, our model has low specificity than some of the state of the arts techniques, but it has outperformed all in terms of sensitivity. It is worth mentioning that sensitivity is more critical than specificity. Furthermore, our model has outperformed some of the sate of the art techniques in terms of AUROC as well (see Table 3).

**Table 3:** Performance comparison of our model for 41 nucleotide sequences.

Predictor	Dataset(m5c)	Performance			
		$S_n$	$S_p$	Acc	AUROC
Identifying RNA 5-methylcytosine					–
sites^26^ via pseudo					–
nucleotide compositions	H. sapiens	85.00	95.83	90.42	–
iRNA-PseColl^27^	H. sapiens	75.83	79.17	77.50	–
iRNA-PseColl^27^	RMbase database	69.89	99.86	92.37	–
Identifying 5-methylcytosine sites in					–
RNA sequence using composite encoding					–
feature into Chou’s PseKNC^29^	H. sapiens	90.00	96.66	93.33	–
M5C-HPCR^30^	H. sapiens and Met1320	90.83	95.00	92.92	–
DeepMRMP^12^	H. sapiens, M. musculus	47.95	84.69	66.32	–
XGBoost Framework...^68^	H. sapiens	89	82.0	85.50	0.935
Attention based multi label...^69^	H. sapiens	92	78.0	85.00	0.910
Our model	H. sapiens	96.70	90.00	96.21	0.979

**Figure 7:**
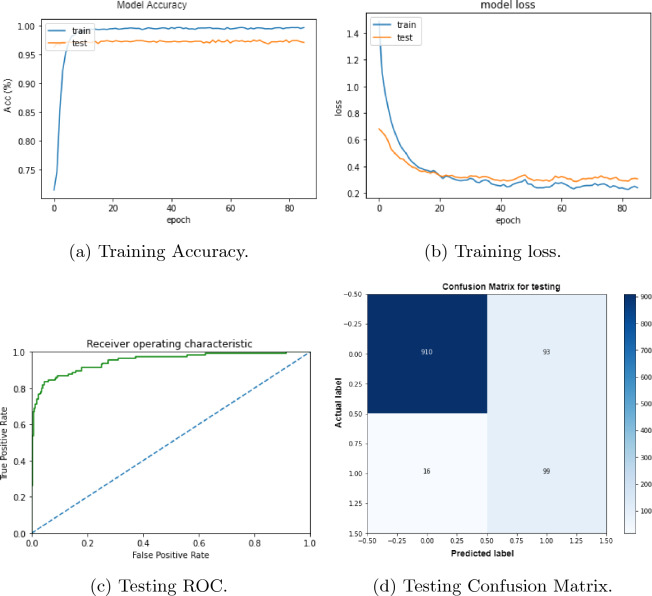
Performance of our model for training/testing data for full length sequences.

The second experiment consists of using the full sequence as input to the model after applying one-hot encoding. Our aim is to perform the experiment in real and most natural way and it is the main focus of this work. In real word the length of the underlying RNA sequences is not always 41 nucleotides and also the methylated cytosine may not be in the center. The training accuracy and loss and testing ROC and confusion matrix are shown in Fig. 7 respectively. It shows the model’s ability to accurately classify the sequence without having a methylated cytosine in the center and considering 41 nucleotide length. Our model achieved accuracy up to 96.10% on test data.

## Conclusions

Accurate prediction of RNA methylated sequences is necessary for understanding the underlying mechanism of the regulation of genes. A convolutional neural network based model was introduced in this work to distinguish methylated sequences from non methylated sequences of human genome. The basic purpose of this research was to build a sequence based deep learning classifier that can m5c RNA methylation using full length sequences. The evaluation of the proposed architecture showed a promising results when compare to the stat-of-the-art techniques.In future we aim to focus on providing a web server for the current work. Furthermore, we want to extend this work to aberrant methylation classification and prediction.

## Data Availability

The dataset used in this study is available in the NAR (Neuclic Acid Researcher) Online [https://academic.oup.com/nar] repository and it is discussed in the section “Dataset”.
